# Supplementary material for: Coordination of capsule assembly and cell wall biosynthesis in Staphylococcus aureus
Source: Nat Commun. 2019 Mar 29;10:1404. doi: 10.1038/s41467-019-09356-x (PMC6441080; doi:10.1038/s41467-019-09356-x)
Supplement: Supplementary file 1 — Supplementary Information [file 41467_2019_9356_MOESM1_ESM.pdf]

Supplementary Information

Coordination of capsule assembly and cell wall biosynthesis in *Staphylococcus aureus*

Rausch *et al.*

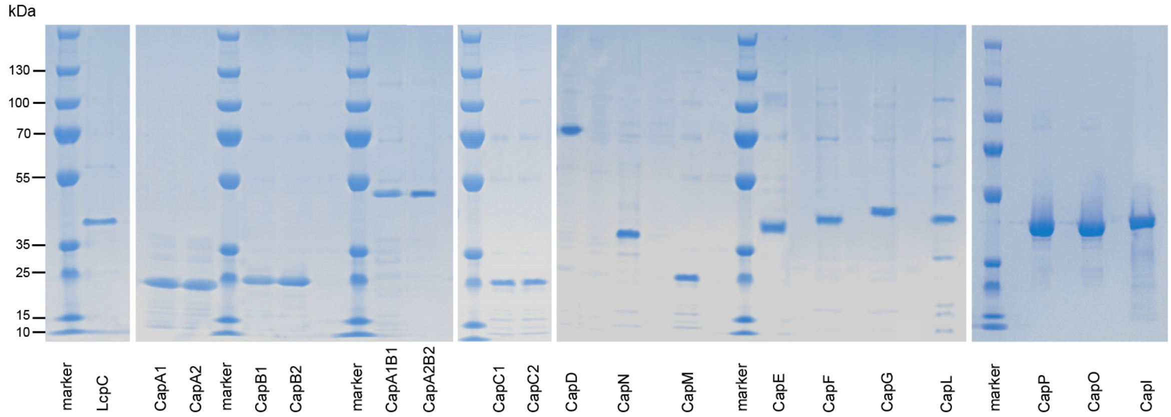

Supplementary Figure 1

SDS-PAGE analysis of purified recombinant His_6-tagged proteins. Protein marker: PageRuler Plus Pre-Stained Protein Ladder (10–250 kDa); Fermentas. Calculated molecular weights: LcpC, 34.7 kDa; CapA1, 24.8 kDa; CapA2, 24.3 kDa; CapB1, 25.3 kDa; CapB2, 25.3 kDa; $CapA1B1_{fus}$, 50.2 kDa; $CapA2B2_{fus}$, 49.7 kDa; CapC1, 28.9 kDa; CapC2, 28.9 kDa; CapD, 69.1 kDa; CapN, 33.7 kDa; CapM, 21.0 kDa; CapE, 38.6 kDa; CapF, 42.2 kDa; CapG, 42.9 kDa; CapL, 46.6 kDa; CapP, 44.3 kDa; CapO, 46.8 kDa; CapI, 42.6 kDa.

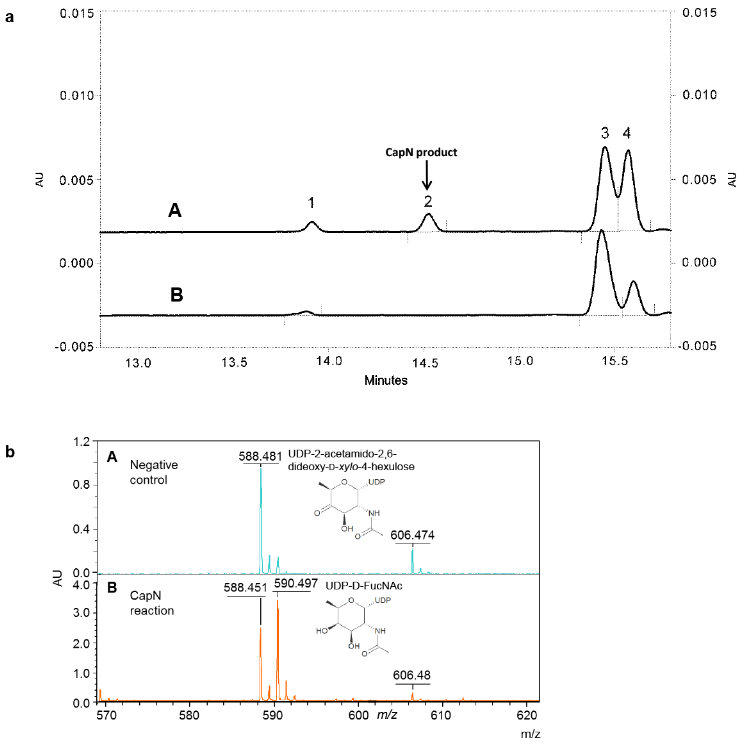

Supplementary Figure 2

CapN enzymatic activity. (a) Capillary electrophoresis (CE) analysis of CapN *in vitro* activity. Purified CapN and PglF were incubated simultaneously in a combined assay in the presence of UDP-GlcNAc and NADPH. (A) PglF/CapN reaction. (B) control reaction containing heat-inactivated CapN. 1, UDP-GlcNAc; 2, UDP-D-FucNAc (CapN product); 3, UDP-2-acetamido-2,6-dideoxy-D-*xylo*-4-hexulose (PglF product); 4, NADP. (b) Mass spectrometric analysis of CapN *in vitro* activity. HPLC-purified sugar nucleotides were analyzed by negative-mode MALDI-TOF mass spectrometry. (A) Mass spectrum of a control reaction containing heat-inactivated CapN. (B) UDP-GlcNAc converted with PglF and CapN. Peaks at *m/z* 588.4 ($[M-H]^-$) correspond to the deprotonated molecular ion of UDP-2-acetamido-2,6-dideoxy-D-*xylo*-4-hexulose (neutral mass 589.3). The peak at *m/z* 590.4 ($[M-H]^-$) corresponds to UDP-D-FucNAc (neutral mass 591.4) and peaks at *m/z* 606.5 ($[M-H]^-$) arise either from UDP-GlcNAc (neutral mass 607.4) or from the hydrated (diol) form of UDP-2-acetamido-2,6-dideoxy-D-*xylo*-4-hexulose. AU, arbitrary units.

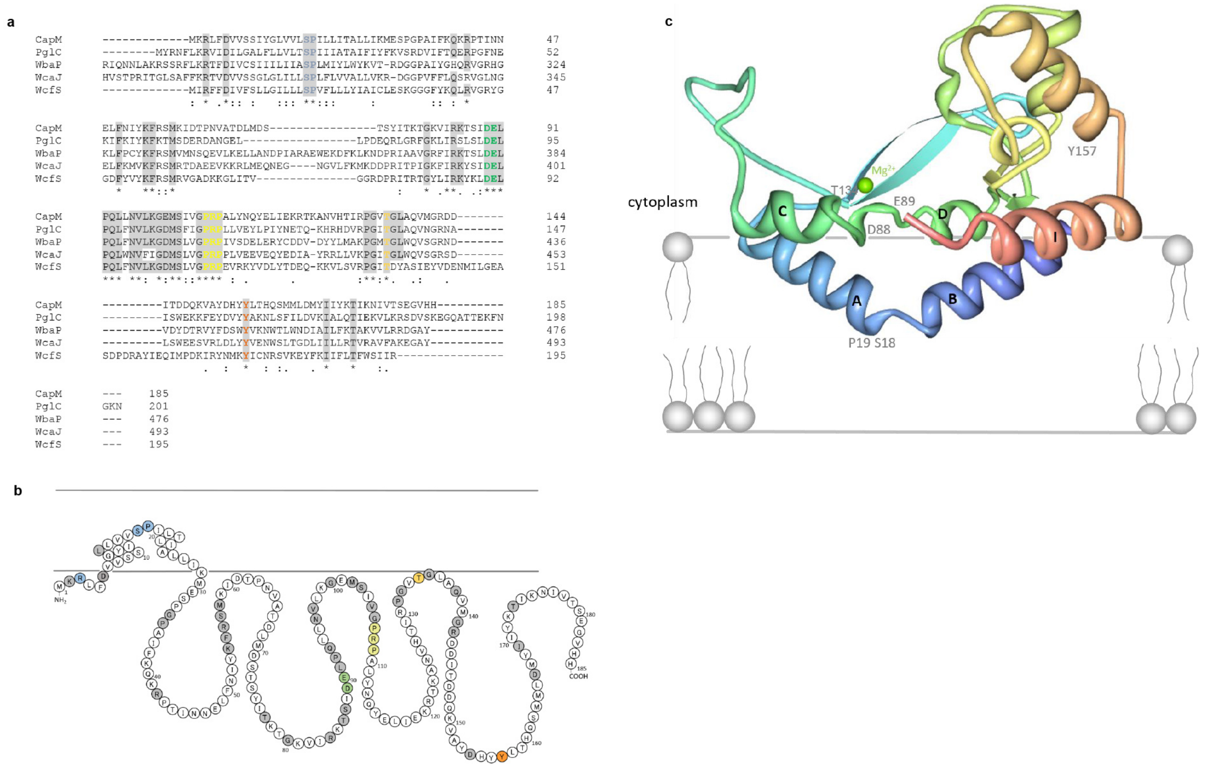

Supplementary Figure 3

Membrane topology and predicted architecture of *S. aureus* CapM. (a) Sequence alignment of CapM (*S. aureus*) with glycosyltransferases WbaP (*Salmonella enterica*), PglC (*Campylobacter concisus*), WcaJ (*Mycobacterium tuberculosis*) and WcfS (*Bacteroides fragilis*). Conserved amino acids are highlighted grey. The highly conserved Tyr157 residue is highlighted orange and Thr134 is highlighted yellow. The catalytic DE dyad is marked green and a strictly conserved PRP motif (107-109) yellow[1]. (b) CapM is anchored in the membrane by a reentrant membrane helix. (c) Structural model obtained by SWISS-MODEL (based on PglC structure of *C. concisus*[1]). The reentrant membrane helix is formed by a helix-break-helix motif of helices A and B (blue) with an interhelix angle of 118° by a Ser-Pro motif (residues 18 and 19). The catalytic DE dyad (residues 88 and 89) is located in the cytoplasm. Mg^{2+} cofactor is highlighted in green.

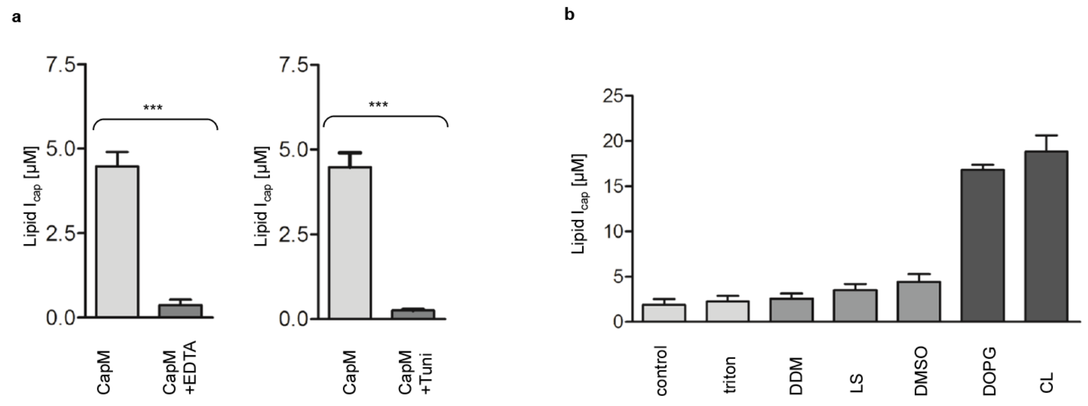

Supplementary Figure 4

CapM enzymatic activity. (a) The impact of $MgCl_2$ (50 mM EDTA) and tunicamycin (Tuni; 200 µM) on CapM *in vitro* activity was tested in the presence of DMSO. (b) Impact of detergents and anionic lipids on CapM *in vitro* enzyme activity. Triton, triton X-100; DDM, *n*-dodecyl-β-D-maltoside; LS*,* *n*-lauroyl sarcosine; DMSO, dimethylsulfoxide; DOPG, dioleoylphosphatidylglycerol (9 nmol); CL, cardiolipin (20 nmol). Experiments were performed in triplicate. The error bars represent the ± SD from three biological replicates. Statistical significance was analyzed by an unpaired *t*-test (****p* < 0.005).

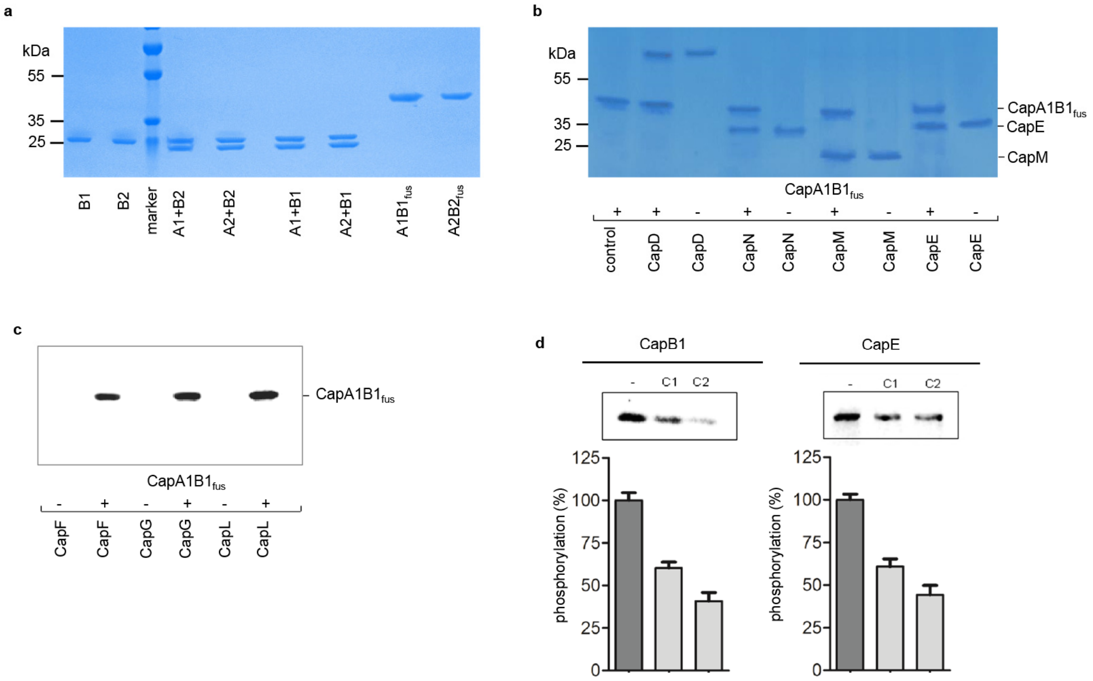

Supplementary Figure 5

CapA1B1 phosphorylation and CapC mediated dephosphorylation. (a) Representative SDS page of CapAB phosphorylation analysis. (b) Representative SDS page of phosphorylation target protein analysis. (c) Analysis of CapA1B1 mediated phosphotransfer to CapF, CapG and CapL by SDS-PAGE and phosphoimaging. (d) The phosphatases CapC1 and CapC2 catalyze the dephosphorylation of target proteins CapB1 and CapE. Experiments were performed in triplicate. The error bars represent the ± SD from three biological replicates.

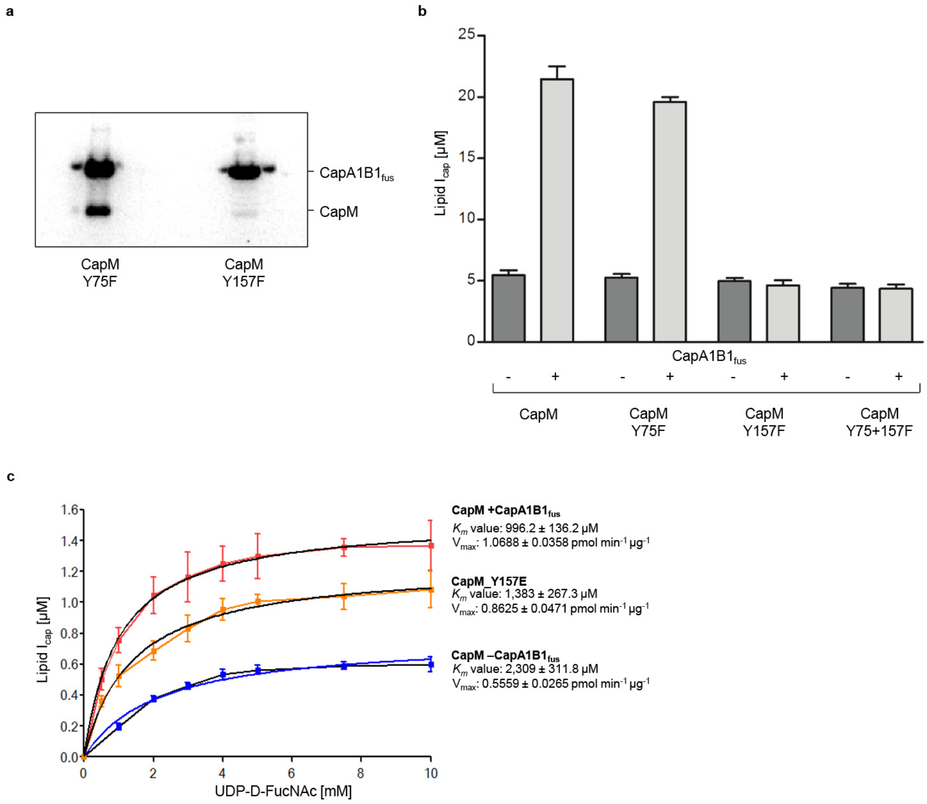

Supplementary Figure 6

Identifying Tyr157 as regulatory phosphorylation site on CapM. (a) CapM mutant proteins carrying Tyr to Phe exchanges in position 75 and/or 157 were assayed for GT activity in the presence of either active CapA1B1 (light grey) or heat-inactivated CapAB (dark grey) in DMSO. Experiments were performed in triplicate. The error bars represent the ± SD from three biological replicates. (b) Determination of K_m and V_{max} values of CapM in the presence (red), absence (blue) of CapA1B1 and of a CapM phosphomimetic variant (Tyr157Glu, orange). (c) CapA1B1 *in vitro* kinase assay with CapM Y75F and CapM Y157F. Phosphotransfer was analyzed by SDS-PAGE and phosphoimaging.

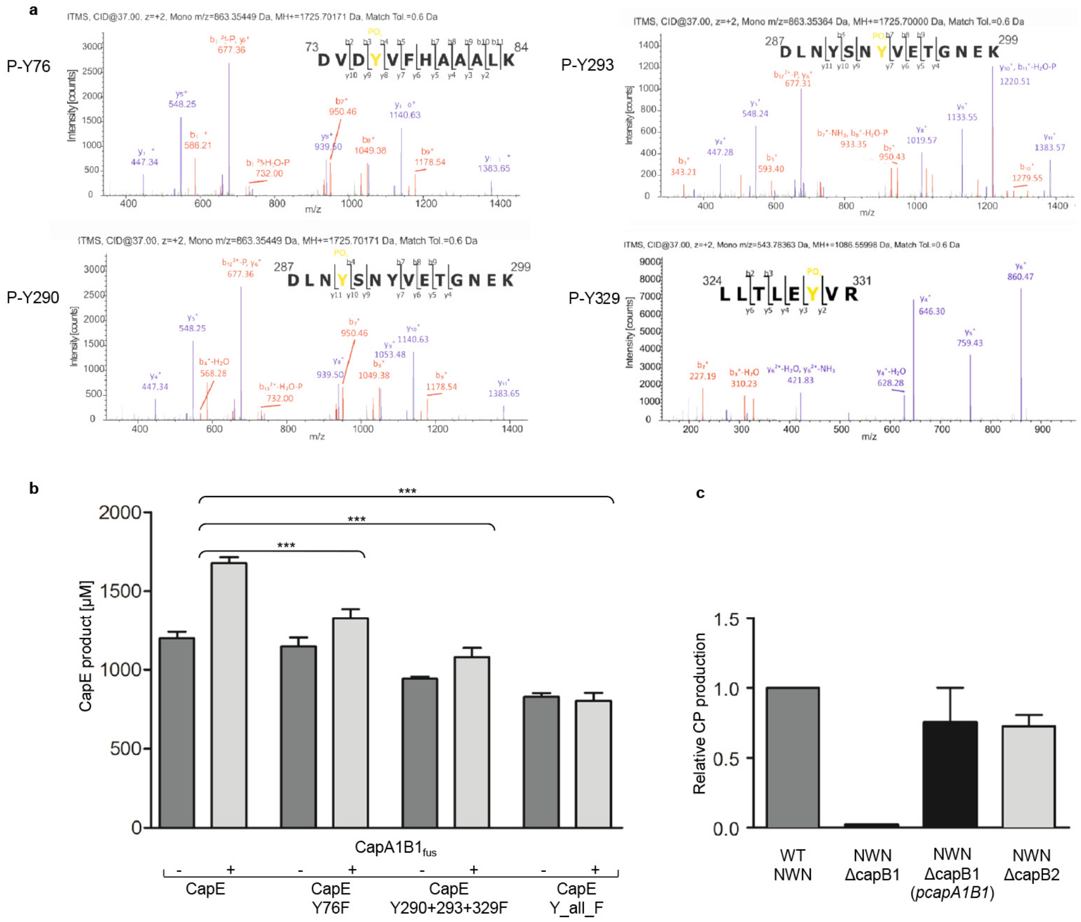

Supplementary Figure 7

CapAB positively controls CapE enzymatic activity. (a) Representative LC/MS spectrum for identification of CapAB phosphorylation sites on CapE. (b) Site-directed mutagenesis of CapE tyrosine phosphorylation sites. CapE mutant proteins carrying Tyr to Phe exchanges were assayed for dehydratase activity in the presence of either active CapA1B1$_{fus}$ (light grey) or heat-inactivated CapA1B1$_{fus}$ (dark grey) using CE detection. Experiments were performed in triplicate, with duplicate measurements. The error bars represent the ± SD from three biological replicates. Statistical significance was analyzed by an unpaired *t*-test (***$p < 0.005$). (c) ELISA-based quantification of CP production by *S. aureus* Newman (NWN) wildtype compared to Δ*capB1,* Δ*capB2* deletion mutants and Δ*capB1* mutant complemented *in trans.* Experiments were performed in triplicate. The error bars represent the ± SEM from three biological replicates.

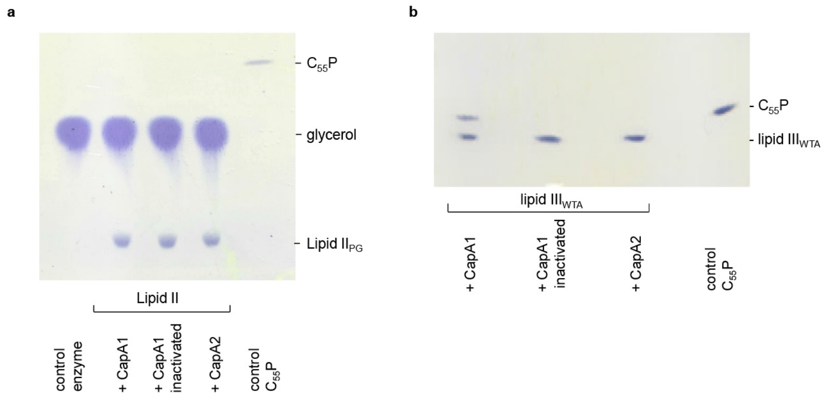

Supplementary Figure 8

The ultimate PG precursor lipid II is no phosphodiesterase substrate for CapA1. (a) Purified lipid II (2 nmol) was incubated with CapA1, CapA2 and heat-inactivated CapA1 (4 µg each). Reaction mixtures were extracted with BuOH/PyrAc and analyzed by TLC and PMA staining. Purified $C_{55}P$ was used as a migration control. (b) The WTA precursor lipid III ($C_{55}PP$-GlcNAc) (2 nmol) is a CapA1 substrate.

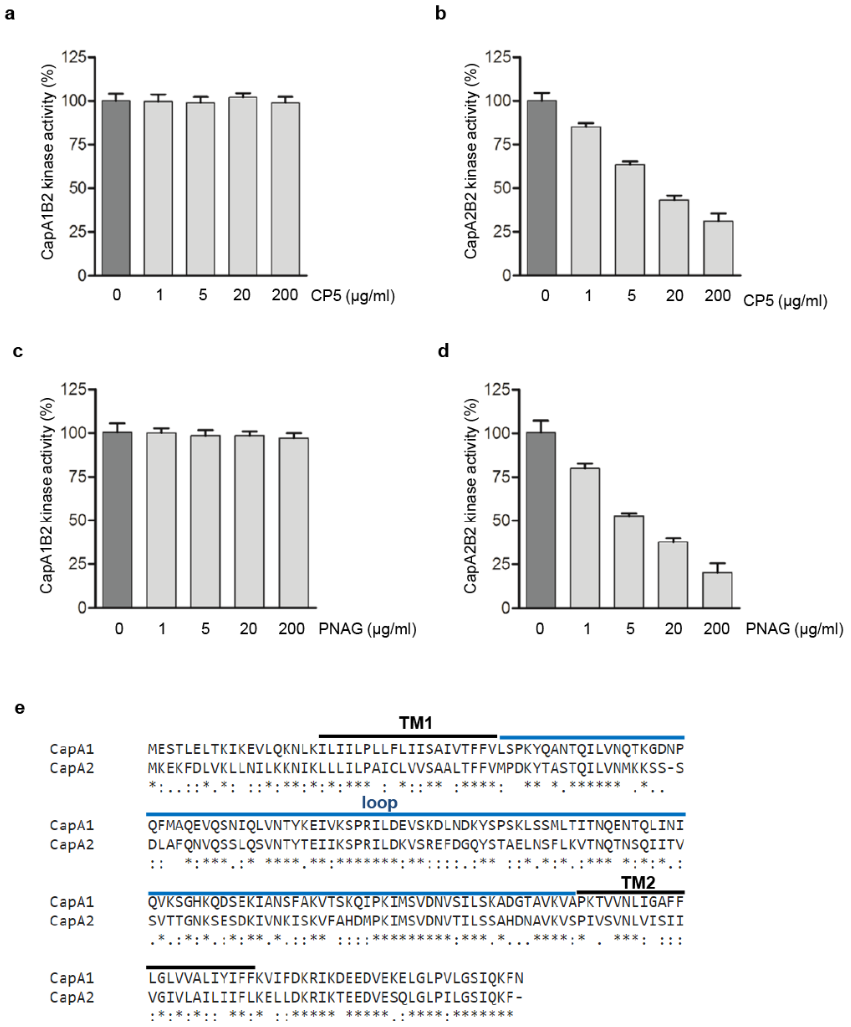

Supplementary Figure 9

CapA2 senses extracellular polysaccharides. CapA2-mediated activation of CapB2 is inhibited by purified polysaccharides CP5 (b) and PNAG (d) in a concentration-dependent manner. In contrast, CapA1-mediated activation of CapB2 is neither affected in the presence of CP5 (a), nor in the presence of PNAG (c). Experiments were performed in triplicate. The error bars represent the ± SD from three biological replicates. (e) Sequence alignment of CapA1 and CapA2 of *S. aureus* Newman. The extracellular loop is highlighted blue.

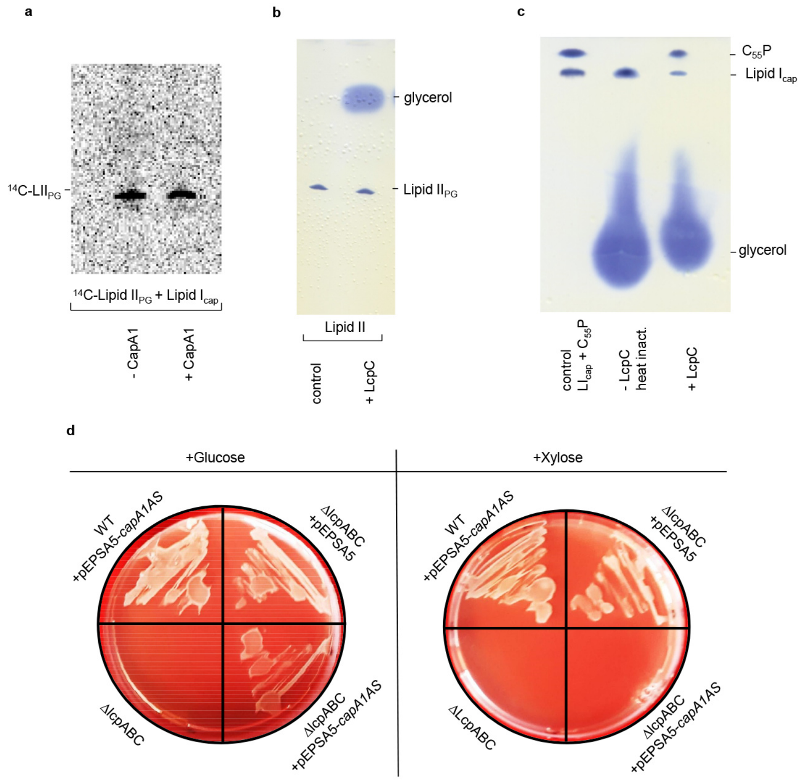

Supplementary Figure 10

The ultimate PG precursor lipid II is no phosphodiesterase substrate for LcpC. (a) CapA1 alone does not catalyze CP-to-PG attachment. Lipid I$_{cap}$ and [^{14}C]lipid II$_{PG}$ were incubated in the absence or presence of CapA1 and reactions were analyzed by TLC and phosphoimaging. (b) Purified lipid II$_{PG}$ (2 nmol) was incubated with LcpC (4 µg). Purified lipid II$_{PG}$ was used as control. (c) Lipid I$_{cap}$ is a phosphodiesterase substrate for LcpC. LcpC alone is able to catalyze cleavage of lipid I$_{cap}$ releasing C$_{55}$P (lane 3) compared to the heat-inactivated control (lane 2). Reaction mixtures were extracted with BuOH/PyrAc and analyzed by TLC and PMA staining. Purified lipid I$_{cap}$ and C$_{55}$P were used as migration controls. (d) Antisense-RNA mediated depletion of CapA1 in a *S. aureus* Newman Δ*lcpABC* mutant is lethal. (right) Expression of a pEPSA5-*capA1AS* encoded *capA1* antisense fragment in a *S. aureus* Newman Δ*lcpABC* mutant induced with 500 mM xylose lead to growth inhibition whereas a wildtype (WT) strain was able to grow. Of note, a Δ*lcpABC* mutant harbouring the empty vector (pEPSA5) as a control grew under similar conditions. (left) *S. aureus* Newman Δ*lcpABC* harbouring pEPSA5 or pEPSA5-*capA1AS* grew under non-inducing conditions (2.5% glucose). All strains where grown on TSA-agar plates containing 34 µg ml^{-1} chloramphenicol supplemented with either 2.5% glucose or 500 mM xylose. Strains lacking the pEPSA5 mediated chloramphenicol resistance were not able to grow. Pictures were taken against a red background.

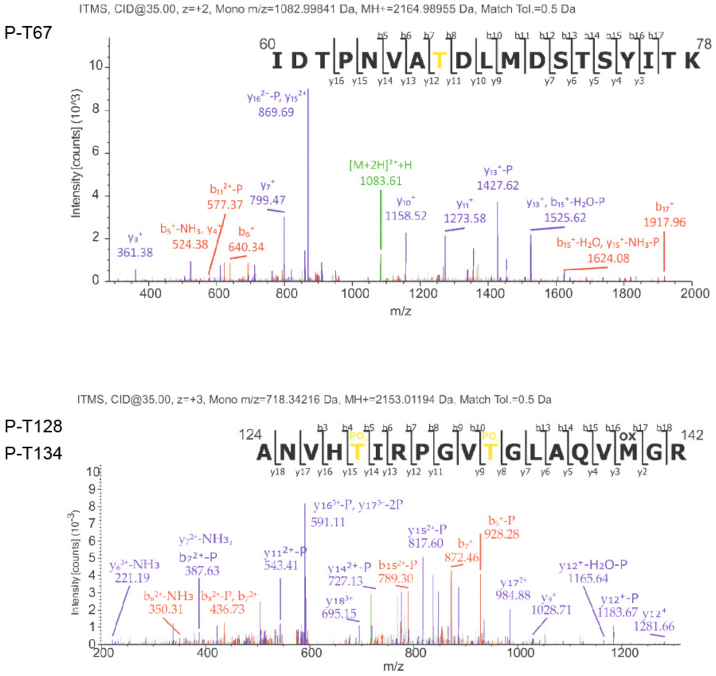

Supplementary Figure 11

Representative LC/MS spectrum for identification of PknB phosphorylation sites on CapM.

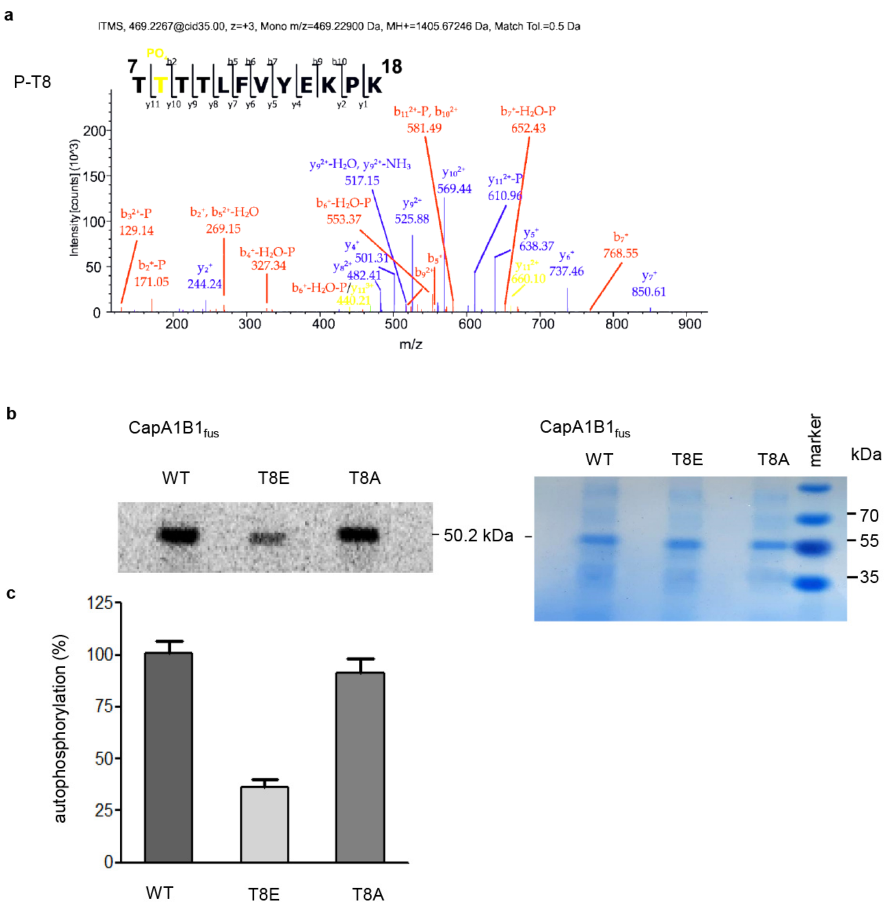

Supplementary Figure 12

PknB negatively controls CapB1 kinase activity. (a) Representative LC/MS spectrum for identification of PknB phosphorylation sites on CapB1. (b) Analysis with loading controls (right) and (c) quantification of the autophosphorylation of a CapA1B1$_{fus}$_T8E-phosphomimetic and a CapA1B1$_{fus}$_T8A-phosphoablative variant compared to the wild-type protein. Experiments were performed in triplicate. The error bars represent the ± SD from three biological replicates.

Supplementary Table 1. Primers used in this study

Primer	Sequence (5′→3′)*
*capA1*_F	GCGCGGCTAGCATGGAAAGTACATTAGAATTAAC
*capA1*_R	GCGCGCTCGAGTTAATTAAATTTTTGAATTGAACCC
*capA2*_F	GCGCGGCTAGCATGAAAGAAAAGTTTGATTTAGTAA
*capA2*_R	GCGCGCTCGAGTTAAAATTTTTGTATTGAACCTAAAA
*capB1*_F	GCGCGGCTAGCATGCTACTTATGTCAAAAAAGGA
*capB1*_R	GCGCGCTCGAGTTATTCATCTCCATAATAGTGAT
*capB2*_F	GCGCGGCTAGCATGACGAATACACGAAGAAGTA
*capB2*_R	GCGCGCTCGAGTCATTCATCAGTCCCATAATATG
*capC1*_F	GCGCGGCTAGCATGAATAAGTATGATTGATATTCAT
*capC2*_R	GCGCGCTCGAGTTATAACCCAAACCATCTTTTCT
*capA1B1fus*_mut_F	GATTGCCTGTATTGGGTTCAATTCAAAAATTTAAT**GCA**GGATGGTTGCTACTTAT
*capA1B1fus*_mut_R	ATAAGTAGCAACCATCC**TGC**ATTAAATTTTTGAATTGAACCCAATACAGGCAATC
*capA2B2fus*_mut_F	AGGATTACCTATTTTAGGTTCAATACAAAAATTT**TA**TTTACGAGGAATTACCATG
*capA2B2fus*_mut_R	CATGGTAATTCCTCGTAAA**TA**AAATTTTTGTATTGAACCTAAAATAGGTAATCCT
*capA1B1fus*_mutT8E_F	GGAAAATACG**GAA**ACAACACTATTTG
*capA1B1fus*_mutT8E_R	TTTTTTGACATAAGTAGCAAC
*capA1B1fus*_mutT8A_F	GTTTTTCATATACAAATAGTGTTGTTGCCGTATTTTCCTTTTTTGACATAAGTAG
*capA1B1fus*_mutT8A_R	CTACTTATGTCAAAAAAGGAAAATACGGCAACAACACTATTTGTATATGAAAAAC
capA1As_F	GCGCGCGAGCTCTATATAAGCGCGACAACTAATC
capA1As_R	GCGCGCGTCGACAAACTGTAGTGAATCTAATCGG
*capE*_mutY76_F	GCGAGATGTTGA**TTT**CGTATTCCATGCAGCAGC
*capE*_mutY76_R	GCTGCTGCATGGAATACG**AAA**TCAACATCTCGC
*capE*_mutY290/293_F	TACCGGTTTCAAC**AAA**ATTACT**AAA**ATTTAAATCTCTGGAGTCTGCCGGCA

*capE*_mutY290/293_R	TGCCGGCAGACTCCAGAGATTTAAAT**TTT**AGTAAT**TTT**GTTGAAACCGGTA
*capE*_mutY329_F	TTATAATCATTCAATTCGTTTCTAAC**AAA**TTCTAGTGTTAAAAGTTTTTCTTTTATCTCTTC
*capE*_mutY329_R	GAAGAGATAAAAGAAAAACTTTTAACACTAGAA**TTT**GTTAGAAACGAATTGAATGATTATAA
*capI*_F	AAAAGGATACTTAATCATATGAG
*capI*_R	TAGTCTACCTCCTCGAGAAATT
*capL*_F	AAGGACGTCGCTAGCATGAGTG
*capL*_R	ATCGTCGACCTTCAACAGATTGTAATAC
*capM*_F	GCGCGGCTAGCATGAAGCGATTATTCGATGTAG
*capM*_R	GCGCGCTCGAGTTAGTGATGCACACCTTCTGA
*capM*_mutY75_F	ATTTAATGGATTCAACATCGTA**TTT**AACAAAGACAGGGAAGGTCAT
*capM*_mutY75_R	ATGACCTTCCCTGTCTTTGTT**AAA**TACGATGTTGAATCCATTAAAT
*capM*_mutY157_F	GTAGCGTATGATCATTAT**TTC**TTAACACATCAATCTATGAGGG
*capM*_mutY157_R	CCCTCATAGATTGATGTGTTAA**GAA**ATAATGATCATACGCTAC
*capN*_F	GTGCAGCTAGCATGAGAAAAAATATT
*capN*_R	AATAGATCTCGAGTGCCTTATCTTTG
*lcpC*_F	GCGCGGCTAGCATGAAACGTTCGTCTAAAAGTAAA
*lcpC*_R	GCGCGCTCGAGCTACTCTAGATTATCTTTTAATAAC

*Restriction sites are underlined, nucleotide exchanges are indicated in bold.

Supplementary Table 2. Plasmids used in this study

Plasmid	Description*	Source/Reference
pET21b	*E. coli* expression vector, C-terminal His_6-tag, Amp^R	Novagen
pET21b-*capI*	pET21b containing *capI* via *Nhe*I/*Xho*I	This work
pET21b-*capL*	pET21b containing *capL* via *Nhe*I/*Sal*I	This work
pET24a	*E. coli* expression vector, C-terminal His_6-tag, Amp^R	Novagen

pET24a-*capD*	pET24a containing *capD* via *Nhe*I/*Xho*I	[2]
pET24a-*capN*	pET24a containing *capN* via *Nhe*I/*Xho*I	This work
pET28a	*E. coli* expression vector, N-terminal His_6-tag, Kan^R	Novagen
pET28a-*capA1*	pET28a containing *capA1* via *Nhe*I/*Xho*I	This work
pET28a-*capA2*	pET28a containing *capA2* via *Nhe*I/*Xho*I	This work
pET28a-*capB1*	pET28a containing *capB1* via *Nhe*I/*Xho*I	This work
pET28a-*capB2*	pET28a containing *capB2* via *Nhe*I/*Xho*I	This work
pET28a-*capA1B1fus*	pET28a derivative for expression of a N-terminally His_6-tagged CapA1B1 fusion protein	This work
pET28a-*capA2B2fus*	pET28a derivative for expression of a N-terminally His_6-tagged CapA2B2 fusion protein	This work
pET28a-*capA1B1*fus_T8A	pET28a-*capA1B1fus* derivative for expression of $CapA1B1_{fus}$_T8A	This work
pET28a-*capA1B1fus*_T8E	pET28a-*capA1B1fus* derivative for expression of $CapA1B1_{fus}$_T8E	This work
pET28a-*capM*	pET28a containing *capM* via *Nhe*I/*Xho*I	This work
pET28a-*capM*_mut1	pET28a-*capM* derivative for expression of CapM_Y75F	This work
pET28a-*capM*_mut2	pET28a-*capM* derivative for expression of CapM_Y157F	This work
pET28a-*capM*_mut3	pET28a-*capM* derivative for expression of CapM_Y[75,157]F	This work
pET28a-*lcpC*	pET28a containing *lcpC* (SA2103) via *Nhe*I/*Xho*I	This work
pET28a-*pglF*	pET28a containing truncated *C. jejuni pglF* via *Nde*I/*Xho*I	[2]
pET5F1.1	pET24a containing *capF* via *Xho*I/*Nhe*I	[3]
pET52b	*E. coli* expression vector, N-terminal Strep-tag, Amp^R	Novagen
pET52b-*pknB*	pET52b containing *pknB* via *Bam*HI/*Sac*I	[4]
pKBK4	pET24a containing capO via *Xba*I/*Eco*RI	[5]
pKBK10	pET24a containing capP via *Hind*III/*Eco*RI	[5]
pKBK6a	pET24a containing *capG* via *Xba*I/*Eco*RI	[3]
pKBK50d	pET24a containing *capE* via *Xho*I/*Nhe*I	[3]
pKBK50d_mut1	pKBK50d derivative for expression of CapE_Y76F	This work

pKBK50d_mut2	pKBK50d derivative for expression of CapE_Y[290,293,329]F	This work
pKBK50d_mut3	pKBK50d derivative for expression of CapE_Y[76,290,293,329]F	This work
pCU1	*E. coli–S. aureus* shuttle plasmid, Amp^R, Cm^R	[6]
pCapA1	pCU1 harbouring *capA1*	[7]
pEPSA5	E. coli-S. aureus shuttle plasmid, Amp^R, Cm^R	[8]
pEPSA5-*capA1AS*	pEPSA5 containing *capA1* antisense fragment via *Sac*I/*Sal*I	This work
pCapA1B1	pOS1-Plgt harbouring *capA1B1* fusion fragment via *Nde*I/*Xho*I	This work

*Resistance marker: Amp^R, ampicillin; Cm^R, chloramphenicol; Kan^R, kanamycin.

Supplementary Methods

ESI-TOF mass spectrometric analysis of lipid intermediates. Electrospray. MS spectra were recorded on a micrOTOF-Q quadrupole time of flight instrument (Bruker Daltonics) working in negative mode. Samples were infused at 0.05 ml h^{-1}, (in methanol-chloroform, 1:1). Signals from the $[M-H]^-$ ions were accompanied in all cases by those from the monosodium adduct and those from the monopotassium adduct.

Identification of phosphorylation sites by nanoLC-MS/MS. *In vitro* phosphorylation of recombinant His_6-tagged proteins, followed by SDS-PAGE separation with omission of radiolabeled ATP from the reaction mixture. For peptide preparation, protein bands were excised from the Coomassie-stained polyacrylamide gels and subjected to tryptic in-gel digestion[9,10]. In brief, proteins were reduced with 20 mM DTT, slices were washed with 50 mM ammonium bicarbonate, and proteins were alkylated with 40 mM iodoacetamide. The slices were washed again and dehydrated with acetonitrile. Slices were dried in a vacuum concentrator and incubated with 400 ng sequencing grade trypsin at 37 °C overnight. The peptide extract was dried in a vacuum concentrator and stored at -20 °C. Dried peptides were dissolved in 10 µl 0.1% formic acid (solvent A), and aliquots (1 µl) were injected onto a C18 trap column (20 mm x 100 µm, NanoSeparations). Bound peptides were eluted onto a C18 analytical column (200 mm x 75 µm, NanoSeparations). Peptides were separated during a

linear gradient from 0% to 55% solvent B (80% acetonitrile, 0.1% formic acid) within 40 min at a flow rate of 400 nl min^{-1}. The nanoHPLC was coupled online to an LTQ Orbitrap Velos mass spectrometer (Thermo Fisher Scientific). Peptide ions between 395 and 1800 *m/z* were scanned in the orbitrap detector with a resolution of 30,000 (maximum fill time 400 ms, AGC target 10^6). The 25 most intense precursor ions (threshold intensity 5000) were subjected to collision induced dissociation and fragments were analyzed in the linear ion trap. Fragmented peptide ions were excluded from repeat analysis for 15 s.

Raw data processing and analysis of database searches were performed with Proteome Discoverer software version 1.40.288 (Thermo Fisher Scientific). Peptide identification was done with an in house Mascot server version 2.3 (Matrix Science Ltd, UK). MS2 data was searched against *S. aureus* N315 NCBInr (release 20130323) and *E. coli* sequences from SwissProt (release 2013_03). Precursor ion *m/z* tolerance was 8 ppm, fragment ion tolerance 0.6 Da, b- and y-ion series were included. Semitryptic peptides with up to one missed cleavage were searched. The following dynamic modifications were set: Alkylation of Cys by iodoacetamide or acrylamide, phosphorylation (Ser, Thr, Tyr), and oxidation (Met). The PhosphoRS3.0 node was used for scoring of the phosphosite assignment[11]. Mascot results from searches against SwissProt were sent to the percolator algorithm[12] version 2.04 as implemented in Proteome Discoverer 1.4.

***In vitro* lipid II synthesis**. Synthesis and purification of lipid II was performed using membranes of *Micrococcus luteus* as described[13–15]. In short, membrane preparations (200 µg protein) were incubated in the presence of purified substrates, 5 nmol undecaprenylphosphate ($C_{55}P$), 50 nmol UDP-MurNAc-pp and 50 nmol [^{14}C]-UDP-GlcNAc in 60 mM Tris-HCl, 5 mM $MgCl_2$, pH 7.5, and 0.5% (w/v) Triton X-100 in a total volume of 50 µl for 1 h at 30 °C. Bactoprenol containing products were extracted with the same volume of butanol/pyridine acetate (2:1; vol:vol; pH 4.2) and analyzed by TLC using phosphomolybdic acid (PMA) staining. For synthesis of higher quantities of lipid II the assay was scaled up and purification was performed as described[16]. Reaction mixtures were incubated for 4 h at 30 °C, and lipids were extracted with the same volume of BuOH/PyrAc. Purification of lipid II was performed on a DEAE-cellulose column (0.9 × 25 cm, DEAE SS-Typ; Serva) and eluted in a linear gradient from chloroform–methanol–water (2:3:1) to chloroform–methanol−30 mM ammonium bicarbonate (2:3:1).

Construction of cap deletion mutants. Tn mutations in the *cap* genes (*capA2, capB2, capA1 and capB1*) were transduced from USA300 mutants into Streptomycin-resistant strain Newman with phage 80 as described by Foster[17] with selection on erythromycin (5 µg ml^{-1}) plates. The mutations were confirmed by PCR, antibiotic resistance, and hemolytic phenotype, and CP5 production was assessed by colony immunoblot[18] and ELISA inhibition assays[19] CP5 was purified from *S. aureus* as described previously[20].

Supplementary References

1. Ray, L. C. *et al.* Membrane association of monotopic phosphoglycosyl transferase underpins function. *Nat. Chem. Biol.* **14**, 538–541 (2018).
2. Li, W. *et al.* Analysis of the Staphylococcus aureus capsule biosynthesis pathway in vitro: Characterization of the UDP-GlcNAc C6 dehydratases CapD and CapE and identification of enzyme inhibitors. *Int. J. Med. Microbiol. IJMM* **304**, 958–969 (2014).
3. Kneidinger, B. *et al.* Three Highly Conserved Proteins Catalyze the Conversion of UDP-N-acetyl-d-glucosamine to Precursors for the Biosynthesis of O Antigen in Pseudomonas aeruginosaO11 and Capsule in Staphylococcus aureus Type 5 IMPLICATIONS FOR THE UDP-N-ACETYL-l-FUCOSAMINE BIOSYNTHETIC PATHWAY. *J. Biol. Chem.* **278**, 3615–3627 (2003).
4. Hardt, P. *et al.* The cell wall precursor lipid II acts as a molecular signal for the Ser/Thr kinase PknB of Staphylococcus aureus. *Int. J. Med. Microbiol.* **307**, 1–10 (2017).
5. Kiser, K. B. & Lee, J. C. Staphylococcus aureus cap5O and cap5P genes functionally complement mutations affecting enterobacterial common-antigen biosynthesis in Escherichia coli. *J. Bacteriol.* **180**, 403–406 (1998).
6. Augustin, J. & Götz, F. Transformation of Staphylococcus epidermidis and other staphylococcal species with plasmid DNA by electroporation. *FEMS Microbiol. Lett.* **66**, 203–207 (1990).
7. Jansen, A. *et al.* Production of capsular polysaccharide does not influence Staphylococcus aureus vancomycin susceptibility. *BMC Microbiol.* **13**, 65 (2013).
8. Forsyth, R. A. *et al.* A genome-wide strategy for the identification of essential genes in Staphylococcus aureus. *Mol. Microbiol.* **43**, 1387–1400 (2002).
9. Rosenfeld, J., Capdevielle, J., Guillemot, J. C. & Ferrara, P. In-gel digestion of proteins for internal sequence analysis after one-or two-dimensional gel electrophoresis. *Anal. Biochem.* **203**, 173–179 (1992).

10. Jeno, P., Mini, T., Moes, S., Hintermann, E. & Horst, M. Internal sequences from proteins digested in polyacrylamide gels. *Anal. Biochem.* **224**, 75–82 (1995).
11. Taus, T. *et al.* Universal and confident phosphorylation site localization using phosphoRS. *J. Proteome Res.* **10**, 5354–5362 (2011).
12. Käll, L., Storey, J. D., MacCoss, M. J. & Noble, W. S. Assigning significance to peptides identified by tandem mass spectrometry using decoy databases. *J. Proteome Res.* **7**, 29–34 (2007).
13. Umbreit, J. N. & Strominger, J. L. Isolation of the lipid intermediate in peptidoglycan biosynthesis from Escherichia coli. *J. Bacteriol.* **112**, 1306–1309 (1972).
14. Brötz, H. *et al.* Role of lipid-bound peptidoglycan precursors in the formation of pores by nisin, epidermin and other lantibiotics. *Mol. Microbiol.* **30**, 317–327 (1998).
15. Wiedemann, I. *et al.* Specific binding of nisin to the peptidoglycan precursor lipid II combines pore formation and inhibition of cell wall biosynthesis for potent antibiotic activity. *J. Biol. Chem.* **276**, 1772–1779 (2001).
16. Schneider, T. *et al.* In vitro assembly of a complete, pentaglycine interpeptide bridge containing cell wall precursor (lipid II-Gly5) of Staphylococcus aureus. *Mol. Microbiol.* **53**, 675–685 (2004).
17. Foster, T. J. 7.9 Molecular Genetic Analysis of Staphylococcal Virulence. *Methods Microbiol. JK Peter Williams George Acad. Press* **27**, 433–454 (1998).
18. Lee, J. C., Liu, M. J., Parsonnet, J. & Arbeit, R. D. Expression of type 8 capsular polysaccharide and production of toxic shock syndrome toxin 1 are associated among vaginal isolates of Staphylococcus aureus. *J. Clin. Microbiol.* **28**, 2612–2615 (1990).
19. Lee, J. C., Takeda, S., Livolsi, P. J. & Paoletti, L. C. Effects of in vitro and in vivo growth conditions on expression of type 8 capsular polysaccharide by Staphylococcus aureus. *Infect. Immun.* **61**, 1853–1858 (1993).
20. Tzianabos, A. O., Wang, J. Y. & Lee, J. C. Structural rationale for the modulation of abscess formation by Staphylococcus aureus capsular polysaccharides. *Proc. Natl. Acad. Sci.* **98**, 9365–9370 (2001).
